# Supplementary material for: Are carbon emissions trading and green financial instruments synergistic? -Comprehensive quantitative research based on content analysis
Source: PLoS One. 2024 Mar 7;19(3):e0298601. doi: 10.1371/journal.pone.0298601 (PMC10919601; doi:10.1371/journal.pone.0298601)
Supplement: S1 Data — (ZIP) [file pone.0298601.s001.zip › Data/地级市Z_F工作报告.zip!/░▓╗╒/░÷▓║╩╨/└┤╫╘╣1⁄2╓┌║┼í╛┬φ┐╦╩2╛▌═°í┐_2015.docx]

各位代表： 
    现在，我代表市人民政府向大会报告工作，请予审议，并请市政协委员和其他列席人员提出意见。 
    一、攻坚克难，新常态中实现新发展 
    2014年，全市各族人民在市委的坚强领导下，认真贯彻党的十八大、十八届三中、四中全会和习近平总书记系列重要讲话精神，紧紧围绕全面建成小康社会、重返全省第一方阵目标，按照抓好群众路线教育实践活动、全面深化改革、推动经济社会持续健康较快发展“三件大事”的部署要求，统筹稳增长、调结构、促改革、惠民生，主要经济指标增速继续高于全省、领先皖北，地区生产总值、规模以上工业增加值、财政收入等多项指标实现总量进位，经济运行质量效益明显提升，保持了争先进位、赶超跨越的良好势头，经济社会发展在新常态中展现了新姿态。全市实现地区生产总值1108.44亿元，总量前进1位；同比增长10.1%，增速居全省第2位。一、二、三产增加值分别增长5.1%、12%、9.4%，分别居全省第2位、第1位和第3位。固定资产投资1244亿元，增长17.3%。财政收入突破200亿元，达208.4亿元，增长14%。城乡居民人均可支配收入分别达24147元、10511元，增长9.3%、11.8%。社会消费品零售总额481.5亿元，增长13.7%，居全省第1位。居民消费价格涨幅低于省控目标，万元地区生产总值综合能耗、主要污染物减排持续下降。 
    （一）稳增长、调结构，综合实力明显增强。 
    工业经济增势强劲。扎实开展服务企业拓展年活动，深入实施服务企业“百千万”行动，减免抵退企业各类税费36亿元，工业总产值突破2000亿元，工业化率达46%。净增规模以上工业企业138户，实现规模以上工业增加值599.9亿元，增长12.8%，居全省第1位。工业经济效益指数居全省第2。大富重工、中华食品产业园、盛亚环保新型建材等90个重点工业项目开工建设，德豪润达倒装芯片、亚伦电子壁炉、中科电力装备产业园一期、方兴科技电容式触摸屏等62个项目建成投产，一批蚌埠制造整机、整器、整车类终端产品开始生成。新增好思家等3件中国驰名商标。大连后青春工业设计中心落户蚌埠。跻身国家信息消费试点市。五河经济开发区扩区、马城经济开发区更名迁建获批。全面启动8个中小企业产业园建设，建成标准化厂房80.5万平方米。西部城区重点化工企业搬迁选址工作全面完成。 
    现代农业实力提升。成功承办全国粮食高产创建及增产模式攻关推进会、全国农村水利工作会议。粮食总产275.5万吨，实现“十一连丰”。五河现代牧业液奶加工项目建成投产，固镇亿只肉鸡产业化项目新建标准化鸡场25个，怀远万头肉牛项目启动建设。新增亿元以上农产品加工企业10家，新组建农民专业合作社189家、家庭农场150个。土地流转面积增加20万亩。新增设施蔬菜面积1.02万亩。市级现代农业示范区建设工作启动。淮干蚌浮段行蓄洪区调整和建设等工程进度加快。新增有效灌溉面积11.8万亩。实施农村土地整治项目125万亩。解决35.14万农村人口饮水安全问题。减少贫困人口2.3万人。首批46个美好乡村建设重点示范村全面建成并通过验收，第二批20个省级美好乡村中心村建设进展顺利。 
    服务业活力焕发。义乌国际商贸城、国际汽车城等市场集聚效应初显，中华玉博园、汇金商业广场等建成投运，联盟国际家博城、新地城市广场等开工建设，国强路市场、二马路市场等“城中市”搬迁顺利完成。在全省率先启动社区商业便民服务体系建设。皖北徽商物流园建设加快，中储粮蚌埠直属库和粮食一库、三库搬迁全面启动。怀远县成为全省首批电子商务进农村试点县，高新区电商产业园开工建设。龙子湖环境综合治理项目部分建成开放，花鼓灯嘉年华获批4A级景区，万达嘉华、利事环球投入运营。新增限额以上商贸企业130户、服务业企业41户。 
    （二）抓项目、聚要素，有效投入稳步扩大。 
    项目建设提质提效。完成“3461”项目投资780亿元，在建亿元以上项目405个。和鼎子午胎、荣辉动力锂电池等102个项目建成投产，中纺油脂产业园、 MEMS产业化基地等199个项目加速实施，国电二期、方兴科技柔性镀膜等205个项目开工建设。新增谋划项目223个，储备规模超3500亿元。争取中央、省补助资金18.5亿元。 
    招商引资量质同升。出台鼓励以企招商以商招商促进重点产业发展实施意见。成功开展台湾、东莞、泉州系列招商活动，积极参与投洽会、高交会等区域合作交流。着力招大引强，日丰电器电缆、瑞晶手机产业园等130个亿元以上项目落户蚌埠，华夏云谷云计算与智慧产业基地、怡得乐电子等17个10亿元以上项目开工建设。深化与央企省企知名民企合作，中建材金刚玻璃、北京华录电子等9个央企合作项目顺利签约，民企合作新签约项目开工率全省第1。引进市外亿元以上项目资金700亿元、增长27%，实际利用外商直接投资11.36亿美元、增长17%，均居全省前列。 
    融资渠道不断拓宽。完善政府性资金以存促贷机制，引导撬动金融机构加大信贷投放，贷款余额突破千亿，存贷比10年来首超70%。新增贷款191亿元，增长23.5%，增速连续3年保持全省第1。昌盛电子、佳先股份、雪郎生物、昊方机电成功挂牌“新三板”。棚户区改造项目新获国开行授信190亿元，居全省首位。三县农村商业银行挂牌运营。在全省率先开展中小微商贸流通企业无抵押集合信用融资。农村集体土地承包经营权和农民住房抵押贷款试水破题、实现放贷。      （三）强功能、优环境，城乡建设扎实推进。 
    城市空间有效拓展。《蚌埠市城市总体规划(2012-2030年)》顺利获批，规划期末城市人口规模220万人、城市建设用地规模220平方公里，位居全省第3、皖北第1。部分区划调整工作全面完成，市区面积扩大至956.9平方公里。制定《关于以“363”框架为引领推进城市集约发展的指导意见》，着力构建以“三环六轴三带”为核心的城市发展主轴。胜利东路、中环线东段主车道建成通车，黄山大道全线贯通，国道104、206南段和省道307、长淮卫淮河大桥开工建设，市区与三县、凤阳城际公交全覆盖，区域通达能力明显增强。      城区功能逐步完善。继续推进城市大建设，完成投资241亿元，净增31亿元。全力开展征迁拆违攻坚扫尾，市区完成征迁502万平方米、拆违98万平方米，交付净地2.96万亩。启动第二轮棚户区改造项目60个，回迁安置棚改居民2万户。开工建设保障性安居工程31080套，廉租房实物配租4333户。新建、续建朝阳南路、学瀚路等道路39条，市区路网逐步完善。 
    城市管理水平明显提升。引入第三方测评机构，实施文明创建和城市管理动态考评。推行环卫工作属地化、机械化、市场化，城区路面保洁标准不断提高。加大“三车”整治力度，取缔县城客运三轮车。全面清理淮河路等14条主干道和部分区域违法违规广告，广告乱象得到遏制。“三线三边”环境整治、老旧小区生活环境治理扎实推进。 
    生态文明建设力度加大。跻身全国绿色循环低碳交通区域性试点城市、水生态文明试点城市，入选全省生态文明先行示范区。大力实施退网还湖工程，拆除围网2.5万亩。成片造林15.2万亩，建设农田林网77万亩，创建森林长廊示范段129公里。积极推行园林绿化认建认养，新增、改造绿地315万平方米。森林覆盖率提高到17.7%。市民广场、龙子湖东公园起步区建成开放，淮河南岸外滩地整治工程、淮河北岸滨河景观带一期完工。三污厂和怀远、固镇污水处理厂提标改造工程顺利完成。垃圾焚烧发电和餐厨废弃物处置项目前期工作有序推进。实施大气污染防治“百日行动”，开展市区建筑施工扬尘、生活燃煤小锅炉、黄标车淘汰等专项治理，PM10平均浓度从上半年的142微克/m3降到下半年的86微克/ m3。 
    （四）推改革、促创新，发展活力加速迸发。 
    重点领域改革稳步推进。深化行政审批制度改革，市级审批事项由197项减少至45项，为全省最少。优化再造审批流程，审批平均时限比法定时限压缩83.2%。免收17项行政事业性收费，降低13项审批前置环节经营服务性收费，对市、区开发园区工业投资项目免收28项行政事业性收费。在全省率先公布市级涉企收费清单，取消、缓征各类收费34项，降标9项，放开22项。整合工业、自主创新等专项资金，构建“1+2+N”产业发展扶持体系。“蚌采商城”成功上线，在全省首推政府采购电商化。健全政府性债务管理风险预警机制，开展国有资产资源大清查。成为全国首批国库集中支付电子化改革试点城市。在全省率先实施市级层面企业“三证合一”改革，新增注册企业4648户、注册资本总额251亿元，分别增长45%、100%。推进中小学集团化办学，新纳入学校7所。农村土地承包经营权和农村集体土地“三权”确权登记颁证试点、小型水利工程管护体制改革有序推进。 
    自主创新能力不断增强。跻身首批省级创新型试点城市，新认定国家高新技术企业44户，实现高新技术产业产值750亿元，增长30%、居全省第1。战略性新兴产业产值突破600亿元，增长25%。全社会研发投入增幅全省第2，发明专利授权量480件、增长17%。新增省级以上研发平台5个，创建院士工作站2个，引进高层次科技人才团队7个。浮法玻璃微缺陷控制与节能关键技术及产业化应用荣获国家科技进步二等奖，0.33mm超薄玻璃打破国际垄断并稳定量产。国家特种玻璃质检中心获批并向社会开放。 
外向型经济逆势攀升。全省首家蚌埠（皖北）保税物流中心实现当年申报、当年建设、当年获批、当年验收、当年运行。蚌埠（皖北）铁路无水港建成启用。蚌埠新港开通淮河流域首个国际集装箱码头，开启千里淮河集装箱运输新时代，一个现代化淮河大港景象初步呈现。蚌埠玻璃设计院成功收购世界500强圣戈班旗下阿旺西斯公司，丰原集团巴西玉米深加工和匈牙利柠檬酸生产项目开工建设。实现进出口总额20亿美元，增长17%。 
    （五）惠民生、增和谐，群众生活持续改善。 
    民生工程深入推进。投入资金45亿元，33项省级民生工程和12项市级民生工程圆满完成。资助困难家庭学前儿童和贫困大学生834万元，补助“一户多残”家庭388万元，发放高龄老人津贴3168万元、社会办养老服务机构床位补助173万元、城乡居民临时救助884万元、大病救助3800万元，奖补11个创建示范社区550万元。新增环保公交车186辆，发放特殊人群免费乘车补贴1130万元，实行传统节假日公交免费乘坐。奖补新建设施蔬菜基地2000万元。新建改造标准化农贸市场4个、农副产品平价店26个，完成20家农产品进超市、社区便利店工作。投入1351万元，实施环境保护能力建设工程。新建、改造公厕113座、路灯8916盏。2667户以船为家渔民上岸安居。 
    各项事业协调发展。义务教育阶段学校标准化建设达标率95.8%、居全省第1，固镇县、淮上区、禹会区被认定为国家义务教育基本均衡县（区）。北师大蚌埠附属学校开学招生，特教中心新校区投入使用。蚌埠大禹文化产业示范园区被命名为全国文化产业示范园区，填补省内空白。中国社科院淮河流域古代文明研究中心、蚌埠院士工作站成立。成功举办中国？蚌埠花鼓灯艺术交流展演周暨文艺下基层和中国梦？淮河情系列文艺活动。新博物馆、档案馆、规划馆完成部分单元展陈布置工作。以固镇守墓老兵欧兴田为原型创作的电影《生死与共》和电影《中国检察官吴群的故事》在全国公映。“中国好人”、“安徽好人”入选人数分别达90人、36人，位居全省前列。首个媒体APP客户端—“掌握蚌埠”投入运营。市中医院新院区、市疾控中心基本建成，市二院与上海长征医院合作成效初显，市一院与上海复旦大学共建妇女儿童临床中心和附属儿童医院进展顺利。成立市慈善总会，筹集善款3822万元。新增公积金缴存单位160个，覆盖面达87%。企业退休人员计划生育一次性奖励兑现。全民国防教育更加深入，国防后备力量建设扎实有效，蚌埠军分区被省军区评为“军事训练先进单位”。民族宗教、外事、侨务、对台、老年人、残疾人、妇女儿童、青少年等事业不断推进，人防、消防、地震、统计、气象、档案、地方志、双拥、机关事务管理等工作持续加强。荣获第三届省文明城市。成功获得省十四届运动会承办权，将成为除省会合肥外唯一承办两届省运会的城市。 
    社会大局和谐稳定。出台支持企业用工促进稳定就业政策，建成人力资源服务产业园，新增城镇就业8.1万人，创历史新高。企业退休人员养老金、被征地农民基础养老金、城镇职工医保救助金最高支付限额等标准全面提高。深化社区管理体制改革，碧水蓝天社区获“全国优质服务窗口”称号。在全省率先完成行政村换届选举。扎实推进平安蚌埠建设，基本实现主要街区监控设施全覆盖。开展信访积案化解绩效年活动，有效减少社会矛盾。实施“六打六治”打非治违、散装食用油整治等专项行动，加强电梯安全监管，安全生产形势保持稳定。 
    （六）转职能、优服务，政府建设不断加强。深入开展党的群众路线教育实践活动，严格执行中央八项规定和省、市作风建设各项规定，着力解决群众反映强烈的“四风”问题。启动新一轮政府职能转变和机构改革，探索建立大农业、大监管、大流通管理体制。市公共资源交易中心成为国家级标准服务试点。自觉接受人大法律监督和政协民主监督，办理市人大代表议案8件、建议151件，市政协提案355件。接受市人大常委会中小工业企业发展、食品安全专题询问，与市政协就老旧小区整治、地下水资源等开展专题协商。推行全市各预算部门预决算、“三公”经费及专项资金信息公开。全市“三公”经费下降28.8%。正式运行“12345市长热线服务中心”。推进依法行政，强化监察和审计工作，严肃查处一批违纪违法案件。 
    各位代表，过去的一年，我们面临的形势复杂严峻，付出的努力异常艰辛，取得的成绩极为不易，这凝聚着全市上下的心血汗水，见证着方方面面的关心支持。在此，我谨代表市人民政府，向全市人民，向人大代表、政协委员，向离退休老同志，向各民主党派、工商联、无党派人士，向各人民团体，向驻蚌解放军、武警官兵和政法干警，向国家和省驻蚌单位，向所有关心和支持蚌埠发展的各界朋友，表示衷心的感谢和崇高的敬意！ 
    各位代表，回顾总结去年工作，我们也清醒地认识到工作成效还有欠缺，经济社会发展中还存在不少困难和问题。地区生产总值、规模以上工业增加值等没有达到预期目标，客观上有宏观形势复杂多变、经济下行压力的影响，但主要还是我们主观上努力依然不够，办法不多、作风不实的问题不同程度存在，一些能够落实的工作没有真正落实，一些可以办好的事情没有完全办好。我市经济总量还不够大，产业结构不优，部分传统产业产能利用率低，战略性新兴产业规模不大，现代服务业发展不快；经济下行压力较大，企业生产经营困难增多、效益下滑，投资意愿下降，财源培植和城乡居民收入持续增长支撑不够；城市发展空间逐步拉开但尚未形成体系，基础设施和公交、公园、公厕等公共服务设施依然薄弱或质量不高；县域经济发展滞缓，城乡统筹面临制约因素较多；教育、卫生、养老等社会事业发展与群众需求还有差距，社会治理创新任务艰巨，环境保护形势不容乐观；政府职能转变还不到位，“四风”问题依然有隐身变形和反弹回潮可能，等等。对此，我们唯有克服不足、加倍努力、加压奋进。 
    二、砥砺奋进，新机遇中力求新作为 
    2015年，是全面深化改革的关键之年，是全面推进依法治市的开局之年，也是全面完成“十二五”规划的收官之年。世界经济仍处于国际金融危机后的深度调整期，总体复苏疲弱态势难有明显改观。国内经济进入中高速增长的新常态，下行压力加大，问题风险增多。省内区域经济竞争和功能强化角力日趋激烈，争先进位更加艰难。同时，我们也看到，全国经济运行总体保持在合理区间，改革效应不断释放，新的增长动力正在生成。国家继续实施中部崛起、中原经济区、老工业基地等发展战略，特别是实施长江经济带战略，提出发挥合芜蚌自主创新试验区引领作用，将蚌埠港、蚌埠机场纳入综合立体交通走廊，以及省委、省政府出台促进皖北发展“新十条”，对我市都是重大机遇。更为重要的是，全市上下对重返全省第一方阵的认同感、参与度、自信心进一步增强，广大市民热爱蚌埠、发展蚌埠、奉献蚌埠的激情进一步释放。我们相信，有多年持续较快发展的能量积聚，有历次应对经济形势变化的经验积累，有不断推进科学发展的实践积淀，依托良好的区位、交通、产业以及政策等组合优势，完全有可能在区域发展格局中更有作为、更有位置、更有形象。我们将坚定信心，提振精神，知难而进，切实做到适应新常态、掌握新要求、抢抓新机遇、主动新作为、实现新发展，牢牢把握新一轮转型升级、加快发展的主动权。 
    2015年政府工作的总体要求是：认真贯彻党的十八大、十八届三中、四中全会和习近平总书记系列重要讲话精神，坚持稳中求进工作总基调，坚持以全面深化改革为统领，坚持以提高发展质量和效益为中心，把转方式调结构放到更加重要位置，持续聚焦产业发展，持续统筹城乡建设，持续强化创新驱动，持续扩大对外开放，持续加强生态建设，持续保障改善民生，不断提升可持续跨越发展能力，为全面建成小康社会、重返全省第一方阵奠定更加坚实的基础。 
    2015年全市经济社会发展的主要预期目标是：地区生产总值增长9%以上，力争达到9.5%左右；一、二、三产分别增长4%、11.7%和8.6%，规模以上工业增加值增长12.5%；财政收入增长9%；固定资产投资增长15%左右；社会消费品零售总额增长13%；进出口总额增长10%；城乡居民人均可支配收入快于地区生产总值增长；城镇登记失业率、人口出生率、居民消费价格涨幅、单位生产总值能耗、国家要求削减的主要污染物排放均控制在省下达的目标以内。主要经济指标增速继续高于全省、领先皖北。为此，我们将重点做好九个方面的工作： 
    （一）坚持产业升级，更强支撑经济增长。 
注重改造提升传统产业与培育壮大新兴产业联动，承接产业转移与增强内生动力并举，深入实施重点产业发展“5116”工程，强力落实“1+2+N”产业发展扶持政策，握紧拳头，决战工业。开工建设铜铟镓硒薄膜太阳能电池、国方电子新一代LED陶瓷基板等项目，竣工投产方兴科技柔性镀膜、中建材金刚玻璃等项目，延伸电子信息产业链条。加快大富工业智能机器人、丰原生物素、中创钛酸钡陶瓷材料等项目建设，提升高端装备制造、生物医药、新材料等先进制造业水平。继续推动整机、整器、整车类产业链终端、价值链末端产品取得新突破，建成投产中科电力装备产业园二期、新大地新能源客车及改装车、宝岛电动车等项目，力争引进创维电子、心里程电脑等项目。引进自行车及配件项目，筹建自行车产业园。加快实施中华食品产业园、新和纱业天然纤维纱、百特新材料等项目，以高新技术和先进适用技术改造提升食品、纺织、建材等传统产业。深入推进中粮生化、丰原集团、八一化工、天润化工等重点化工企业异地技改、整体搬迁，加快城区存量企业退市进园步伐。 
    健全服务企业常态化、制度化、规范化机制，引导、扶持骨干企业做大做强，优化中小微企业发展环境，着力让市场主体数量多起来、经营活起来、实力强起来。完善经济运行统计分析和监测预警制度，加强煤电油运和资金等生产要素调度衔接，切实解决企业发展中的实际困难。推动环球药业、绿雨种业等企业上市，支持蚌埠玻璃设计院、丰原集团等企业跨区域兼并重组、境外并购和投资合作。净增规模以上工业企业130户以上、产值10亿元以上企业5户，规模以上工业企业总数向1000户冲刺。新增专精特新企业30户、两化融合企业25户，着力培育昊方机电、行星工程等一批细分市场“小巨人”企业。新建中小企业标准化厂房30万平方米，引进孵化企业100家。鼓励企业参与国家、行业标准制定，争创驰名商标2件、省政府质量奖1个。 
    坚持业态融合、布局集聚、功能综合、定位高端，着力增强商贸辐射力、市场凝聚力、物流通达力、旅游吸引力，加快重塑区域商贸重镇步伐。提升义乌国际商贸城运营水平，推进市场区、仓储物流加工区建设。完善国际汽车城服务功能。加快实施鹏欣水游城、文创智慧城等项目。推进徽商物流园、迅捷物流等项目建设，开工建设皖北合一冷链产业综合体、普洛斯仓储等项目。深入推进15分钟便民生活服务圈建设，新增社区便利示范店10个，新建、改造标准化农贸市场4个、平价店15个。加快“新网工程”建设步伐。开展电子商务进农村综合示范，争创省级电子商务示范县（区）1个，支持丰原集团、淮商集团等企业开展特色产品网络销售。建成嘉年华室内水公园，运营喜来登酒店、迎宾馆，加快推进龙子湖环境综合治理、蚌埠旅游集散中心等项目。实现旅游总收入140亿元，增长10%以上。加快4G网络建设与发展。积极发展工业设计、咨询、快递等现代服务业。大力实施养老健康家政、信息、旅游、住房、绿色、教育文化体育等六大领域消费工程，多措并举促进大众消费。净增限额以上商贸企业40户、服务业企业15户。 
    （二）坚持固本强基，更实强化“三农”工作。 
    深入实施农业百佳工程，着力三提狠抓五统，加快转变农业发展方式，不断提升农业产业化、现代化水平，加速农业大市向农业强市迈进。推进粮食生产三大行动和粮食高产创建活动，启动稻茬麦增产工程，建设万亩高产创建示范片31个。实施“菜篮子”提升工程，创建市级蔬菜标准园15个。大力发展畜牧业标准化、规模化健康养殖，开工建设五河现代牧业液态奶二期，完成固镇亿只肉鸡产业化项目标准化鸡场建设、怀远万头肉牛项目核心牛场基础设施建设和种牛引进，规模养殖比重提高3个百分点。每个县实施3个“种养加”、一二三产融合互动的农业产业化项目。完成主要河湖退网还湖，大水面生态养殖面积达10万亩。加快台湾巨力旺食品科技产业园、温州老李集团食品工业园等建设，促进农产品转化增值。争创省级现代农业示范区1个、农业产业化示范区1个，强化市级现代农业示范区建设工作。推进蚌埠国家农业科技园区龙亢核心区“一城五园”建设，提升农业科技创新能力和水平。加快新品种、新技术、新模式、新机具等实用技术推广应用，积极发展代耕代种、专业化统防统治、农机合作社等社会化服务组织。继续推进淮干蚌浮段行蓄洪区调整和建设等重大水利工程建设，提升改造“八小水利工程”，新增有效灌溉面积9万亩，建设旱涝保收田12.08万亩。加大现代农业综合开发力度，完成高标准农田改造29.44万亩。大力发展“放心粮油”和“主食厨房”工程。推行农业标准化清洁生产，完善节水、节肥、节药激励约束机制，筹建市级农产品检测中心。 
    有序有力推进农村综合改革，切实增强农业农村经济发展活力，真正让农业强起来、农村美起来、农民富起来。稳步实施农村集体土地“三权”和承包经营权确权登记颁证工作。推进农民土地承包经营权、农民住房财产权抵押担保贷款试点，探索开展建设用地“增减挂”由整村推进细化到整户推进试点，逐步把沉睡的资产变成流动的资本。鼓励土地经营权向专业大户、家庭农场、龙头企业等新型主体流转，开展适度规模经营，新型主体流转土地比例提升10%。支持农民以经营权入股发展农业产业化经营，探索农民专业合作社开展信用合作、成立农村股份合作社，新增省级农民专业合作社5家。试点农村集体产权制度改革，探索村集体资产运营和收益分配新机制。推进小型水利工程产权制度改革，调动群众建设农田水利积极性。统筹整合涉农资金，增加财政农业支出，提高政策效能。 
    继续推动重大产业项目向县域布局、重大基础设施向县域延伸、优质资源要素向县域集聚，着力激活县域经济，力争增长速度达到全市平均水平。按照“三治三增三提升”要求，深入开展县城规划建设管理提升行动，加快把三县县城发展成为宜居宜业、特色鲜明的小城市。支持每个县集中做大做强2—3个主导产业，以城带产、以产兴城、产城融合发展。加快推动河溜、新集、任桥、曹老集等6个全国重点镇建设，每个县重点支持2—3个中心镇加快发展。新启动20个省级美好乡村中心村建设。 
    （三）坚持全域统筹，更快推进新型城镇化。 
    以规划为引领，以人的城镇化为核心，以提升公共服务、加强基础设施为保障，积极稳妥开展新型城镇化试点工作，优化经济社会发展空间格局。组织实施新版城市总体规划，编制完成综合交通、淮河两岸景观总体概念等规划，积极争取省“多规合一”试点。围绕“363”城市发展主轴，促进公共服务设施向内环集聚、产业配套设施向中环集聚、交通服务设施向外环集聚、商业商务设施向六轴集聚，构建蚌怀、蚌固、蚌五市县统筹发展带，在强化城市建设管理中提升承载力、辐射力，在推进城市功能升级中增强带动力、影响力。持续推进“三权落实”和“五有并轨”，促进基本公共服务常住人口全覆盖和均等化，引导农业转移人口就地就近城镇化，让进城农民搬得来、住得起、留得住。 
    着力增强对内对外通达能力，提升新城综合开发水平，完善老城服务功能配套，促进空间集约、产业集中、人气集聚，做精、做优、做美中心城区。实施城市大建设项目190个，完成投资240亿元左右。谋划争取亳州—蚌埠、蚌埠—淮安客运专线等横向铁路纳入国家“十三五”及铁路发展规划，力争民航机场、水蚌线外迁、中环线贯通、延安路跨河通道等项目取得实质性进展。提升改造国道206、104和省道306、307干线，完善交通服务功能。加快长淮卫淮河大桥建设，打通淮河路、国强路、曹山路等一批断头路，优化市区路网结构。推进高铁东站房、东广场前期工作，进一步完善高铁新区基础设施。健全交通枢纽站所等公共服务功能，大力推进滨湖新区建设。建成姜桥路、货场八路，筹建城南九年一贯制学校，加快城南新区开发建设。同步推进老城改造，完成市区征迁300万平方米，交付净地1.5万亩，实现违建零增长。高效利用国开行授信支持，积极推进货币化、商品房安置，加速实施新一轮棚改，全面完成城市内环以内棚户区和城中村改造任务。开工建设各类保障性安居工程223万平方米，竣工222万平方米，棚改回迁安置2万户，保障房实物配租4535户、租赁补贴1000户。 
    注重建管并举、标本兼治，提高城市管理信息化、精细化、科学化水平，推进文明城市常态化建设，着力提升城市形象和品位。全面治脏。切实解决老旧小区、背街小巷、城乡结合部等重点区域垃圾乱堆乱倒、清洁保洁不到位等问题。采取招标进入、社会定价方式，推进环卫保洁市场化改革。重点治乱。开展无证养犬专项整治，加大对违法搭建、破墙开店、破坏公绿等行为查处力度。加强物业服务行业监管，积极探索形式多样的物业管理模式，加快构建三级物业管理体系。有效提升。建立示范路综合创建机制，建成启用数字化城管，推进城市智能化、精细化管理。分期分批推进老旧小区改造，提高绿化、净化、亮化水平和水电气保障能力。力争跻身全国文明城市提名城市。 
    （四）坚持项目投资，更大发挥拉动作用。 
    严格遵循经济工作项目化、项目工作责任化，全面梳理各类在手、在谈、在建项目，分类分层分阶段细化推进落实，力促项目提质提效。健全重大项目调度机制，建成投运汉景智能显示、万达二期等项目，加速实施蚌埠卷烟厂异地搬迁技改、中恒城市广场（二马路精品城）等项目，开工建设富士成像机器人等项目。运用PPP模式、股权融资等多种方式，吸引社会资本参与实施蚌五高速公路、城市排水及污水处理、曹山森林公园等基础设施项目。新增项目储备规模1万亿元。圆满完成“十三五”规划编制工作。 
    突出市场主体作用，以产业引企业、以技术聚资本、以服务促项目，推动招商引资朝着结构优化、深度拓展、效益提高方向转变，不断提升招商精准度和产业布局集中度。强化产业研究分析、产业链条分解，围绕产业链缺失环节，聚焦终端产品，实施精准招商。围绕“一线四点”（京沪高铁沿线、长三角、珠三角、环渤海、台湾）重点区域开展专题招商、以商招商、以企招商。深化与央企省企民企外企合作，加快214所MEMS传感器、中建材金刚玻璃等项目建设。完善驻点招商，探索招商引资和招才引智相结合新机制。加大招商引资统筹力度，既要立足本位各显神通，更要着眼全局群仙聚会，分县区功能定位，重点围绕1—2个主导产业，开展集中、集约、集群招商。细化落实后续服务、预响应机制，打造项目入驻绿色通道、企业发展黄金通道。深入开展效能评级、效益评估、效果评价，持续提升招商引资质量和效益。实际利用市外亿元以上项目资金750亿元，每个县区至少引进20亿元以上产业类项目1个。 
    坚持政府投资保续建、保重点、保民生，民间投资重引导、重结构、重效益，完善“要素跟着项目走”保障机制，推动有限资源向大产业、大项目倾斜，促进投资稳定增长和结构优化。注重间接融资与直接融资并举、盘活存量和用好增量并重，保持社会融资合理增长，新增贷款冲刺200亿元。加大投融资体制改革力度，有效撬动社会资本参与重点领域建设。健全金融为实体经济服务机制，支持金融机构创新更多、更符合市场经济需求的金融产品和信贷模式。积极争取发行市政债券，完善与央企长期深度战略合作机制。推动凤凰滤清器、中晶光电等企业挂牌新三板，打造蚌埠板块。引进、新设1家股份制银行、证券营业部或保险公司分支机构。开展国家工矿废弃地复垦利用试点，实施工业用地全过程、全要素、全审核管理，用足盘活城镇现有土地资源。优化投资结构，工业投资突破500亿元。 
    （五）坚持改革开放，更好激发竞争优势。 
    坚持问题导向、发展导向、市场导向和民生导向，按照市场要活、创新要实、政策要宽的要求，不断深化重点领域和关键环节改革，让要素活力竞相迸发、制度红利加速释放。认真落实权利平等、机会平等、规则平等改革要求，进一步降低门槛、放宽准入，放手发展民营经济，民营经济占比提高1个百分点。坚持以管资本为主加强国有资产监管，以发展混合所有制为主攻方向，促进各类资本交叉持股、相互融合。完善涉企收费目录清单制度，探索实行“一站式”收费或零收费。落实建筑业、房地产业、金融业和生活服务业等领域“营改增”工作。优化财政运管体制，严格专项资金分类管理、项目支出定期退出，提升资金使用效能、效率、效果。清理、甄别、锁定政府性存量债务，健全考核问责机制，防范化解债务风险。深化预算管理改革，细化预算编制、硬化预算执行，着力年中不追加、年底不结转。启动不动产统一登记工作。持续提升社会化办医、集团化办学质量规模，逐步实现就业、就医、就学等公共服务均等化，让改革春风吹暖百姓身心，让千家万户共享改革福利！ 
    抓住国家扩大内陆开放的有利时机，充分利用两种资源、两个市场，深入实施大通关战略，构建开放经济新格局。强化蚌埠（皖北）保税物流中心运营管理。加大铁海联运推进力度，年货物吞吐量增长30%以上。增开集装箱班轮航线，谋划建设公路无水港、铁路一类口岸、水运二类口岸，争取省级内外贸一体化市场试点。实现进出口22亿美元。引进富士（中国）控股、泰和电路科技等一批外资企业，利用外商直接投资12.5亿美元，增长10%。加快走出去步伐，力争丰原集团境外投资柠檬酸和玉米深加工项目建成投产。积极参与长三角区域分工合作，加大皖北“四化”协调发展先行区建设力度，着力推动淮河生态经济带上升为国家战略。 
    坚持错位发展、差异竞争、优势互动，立足自身的资源禀赋、产业现状、比较优势，提升主导产业集聚度、关联度，统筹推动开发区转型升级。紧抓国家建设长江经济带重大机遇，谋划建设承接产业转移示范区和共建产业园区。优化开发区管理模式和运营机制，探索实施政府雇员制，允许实行企业化管理和绩效考核，推动开发区二次创业。加快物流、商业、医疗、教育等基础设施建设，增强开发区综合配套服务功能。严格开发区土地集约利用评价考核，明确准入门槛，健全退出机制，提高引进项目质量和土地利用率。推动高新区与天河科技园区融合互动，争创国家创新型特色园区。加快经开区现代服务业集聚，服务业增速高于全市平均水平。支持蚌埠铜陵现代产业园建设和发展，工业总产值突破10亿元。 
    （六）坚持创新驱动，更足释放发展活力。 
    厚植创新创业文化，用足用活合芜蚌自主创新试验区和创新型城市平台，健全区域创新体系，激发创新创业活力，以大众创业、万众创新催生发展新动力。加强创新公共服务平台建设，建成运营清华启迪园，推动怀远经济开发区升级省高新技术产业开发区。继续拓展与复旦、合工大等高校产学研用合作，加速清华科技园蚌埠孵化基地、电子信息产业研究院等实体化运作。加大对重点成果转化项目资金扶持力度，鼓励高校和科研院所科研成果在蚌转化。加快科技金融创新园建设，专利权质押贷款突破2亿元。 
    注重创新政策、创新资源、创新人才向企业集聚，将技术研发机构建立在企业，将科技服务覆盖到企业，将创新政策落实到企业，真正使企业成为创新主体。兑现仪器设备加速折旧等激励政策，强化对科技型企业扶持。加快浮法玻璃新技术国家重点实验室、省天然气压缩机工程技术中心建设，攻克一批制约产业发展的关键技术。探索国际科技合作，推进TFT基板玻璃浮法生产等项目落地转化。支持蚌埠玻璃设计院集成创新，全线打通薄膜太阳能电池生产、特种玻璃制造技术等新能源相关产业链，实现新一代铜铟镓硒薄膜太阳能电池全面国产化。新增国家高新技术企业25家，高新技术产业产值冲刺1000亿元，战略性新兴产业产值突破700亿元。发明专利授权量550件。争取获批国家级创新型试点城市。 
    牢固树立人才是第一资源理念，紧紧抓住引进、培养、使用等关键环节，集聚创新创业人才，力争引进一个人才、催生一个产业、带动一批企业。放大人才特区政策效应，继续扩大企业股权和分红激励试点范围，把更多的人才招来留住，把更多知识产权、技术成果转化成实实在在的产业活力，为催生新企业、新产业注入源动力。做强人力资源服务产业园，引进知名人力资源服务企业6家。坚持招才育才并重，加强与清华、复旦等知名高校合作。引导校企合作办学，鼓励高校毕业生留蚌创新创业。培育省115产业创新团队2支。 
    （七）坚持传承引领，更显文化特质魅力。 
    强化对文化价值的深度认同和文化建设的责任担当，以坚定的文化自信、深刻的文化自醒、高度的文化自觉，用蚌埠优秀历史文化根脉滋养城市精神，加快建设特色文化名城。高举中国梦精神旗帜，积极培育和践行社会主义核心价值观，大力弘扬“创新进取、责任担当、务实诚信、合作共赢”的先进思想文化。广泛开展道德实践和志愿服务，营造温馨珠城，淬炼好人蚌埠。大力宣传全国公安机关爱民模范李仁强、司法部二级英模朱儒玲等先进典型，让更多的市民争当为善的行动者。争创未成年人思想道德建设先进城市。积极实施市民素质教育提升工程，深入开展乡风文明“123”创建活动，推动市民革除陋习，做文明有礼蚌埠人。 
    按照公益性、基本性、均等性、便利性要求，全面繁荣发展文化事业，积极构建现代公共文化服务体系，不断满足广大群众日益增长的精神文化需求。加强双墩、大禹等传统特色文化资源的保护、开发和传承、利用，提高蚌埠文化的知名度、美誉度和辨识度。启动花鼓灯艺术申报世界非物质文化遗产工作。推动文艺精品创作，编创《大禹》等精品剧目，拍摄《珠城的传说》等影视作品，记录蚌埠之美、弘扬蚌埠之魂、传播蚌埠之名。大力实施文化惠民工程，开放市少儿图书馆和美术馆，加快推进新文化馆、音乐厅、科技馆规划建设。增设社会科学文学艺术奖。管理使用好乡镇综合文化体育站、社区文化中心和农村文化乐园，让百姓演、百姓看、百姓评、百姓乐，大街小巷都有百姓大舞台。 
    怀着敬畏之心、守望之情、呵护之意，对具有蚌埠印记、蚌埠符号、蚌埠特质的优秀文化进行深度挖掘、高端创意、高位开发，努力把文化资源优势转化为文化产业优势。加快推进国家级大禹文化产业示范园区建设，倾力打造荆涂淮河湾。实施省“861”文化产业项目14个，建成开放白石山佛教文化园，加快推进大明文化产业园、双墩遗址公园、谷阳城遗址公园等项目，提升星宇文化产业园经营规模和水平。促进文化与科技、旅游、金融等深度融合，加速形成创意设计、文化旅游、工业美术等产业集聚区，推动文化产业转型升级。深化文化体制改革，培育壮大蚌埠报业传媒、广播电视传媒、文化演艺等文化企业集团。大力发展动漫数字、玉文化、印刷会展等产业。 
    （八）坚持绿色发展，更美营造生态家园。 
    强力治理雾霾，以更大力度、更实措施、更严问责，全员、全年、全市域强力攻坚大气污染防治，让城市更清新、百姓更舒心。深入推进大气污染防治行动计划，落实大气污染联防联控措施，着力降低空气污染指数，切实改善大气环境质量。坚持疏堵结合，继续推行全市全年全面秸秆禁烧。加快怀远生物质发电项目建设，实现市辖三县生物质发电全覆盖。大力发展清洁能源，抓好机动车污染防治，全面淘汰市区黄标车。加强煤炭消耗总量控制，全面取缔建成区和县城燃煤小锅炉。强化建筑施工和道路扬尘污染治理，推行施工工地沙土物料全苫盖、工地路面全硬化、出入车辆全冲洗、拆除房屋工地全洒水、暂不开工空地全绿化、运输渣土等物料车辆全封闭。加大惩处力度，对违反规定、不达标、不作为的企业停产、车辆停运、工地停工、商贸停业、干部停职。 
    狠抓节能减排，注重源头严防、过程严管、后果严惩，坚持不懈保护生态环境，推动经济社会发展和生态文明建设互动双赢、融合共生。提升污水处理厂运营水平。强化燃煤电厂等企业治污设施运行监管，实施涂山热电、新源热电、华光集团、中联水泥脱硫、脱硝、除尘设施升级改造。组织开展千企万户节能低碳行动，推进循环经济示范县区建设。定期发布节能目标完成情况晴雨表，实施节能目标责任现场评价考核，推行节能减排绩效管理试点。大力开展与农民生产生活密切相关的安全饮水、垃圾污水处理、卫生改厕、河塘沟渠清淤等农村环境综合整治工作，着力解决“上气、下水、循环”难题，推进生产、生活、生态的“三生”良性循环。启动新型电动公交车更替计划，新增纯电动公交车50辆。 
    持续活水增绿，加快建设水在城中、城在林中、城依山、水映城的山水园林城市，让“走千走万不如淮河两岸”再次拨动蚌埠人的心弦。实施最严格水资源管理，着力改善水生态环境。做活水文章，继续推进天河-张公湖等环城水系建设，加大“一河三湖”水环境综合整治力度，让河湖净化、两岸绿化、沿线美化。深入推进千万亩森林增长工程，实施高速公路沿线、城市周边、河湖滩地、农田防护四大绿化提升工程，开展森林城市、森林城镇与森林村庄三大创建活动，成片造林8.06万亩，建设农田林网65万亩。继续深入开展“绿满珠城”行动，新增城镇园林绿化面积340万平方米，改造绿地180万平方米，新建街头绿地（游园）20个，建成城市绿道50公里。积极争创国家园林城市和国家生态文明先行示范区，执着追求净水、蓝天，努力让盎然绿色浸染蚌埠大地。 
    （九）坚持民生优先，更多共享发展成果。 
    群众对美好生活的向往，是我们的奋斗目标；群众的现实需求，是政府的务实重点。我们将投入更多的人力、财力、物力，努力让更多城乡居民学有优教、住有宜居、病有良医、老有善养。认真落实城乡居民养老保险等33项省级民生工程。充分发挥优质教育资源示范辐射作用，推动学前教育、义务教育均衡发展。积极发展职业教育，加快蚌埠职教园区一期建设。开工建设安徽科技学院蚌埠校区。加快推进市三院改扩建和市一院综合病房大楼、市妇女儿童医院项目，启动市二院新院区建设。精心筹备省十四届运动会，启动市体育中心等场馆建设。大力推进全民健身运动，规划建设全民健身场馆，逐步让山上楼下、林中岸边都有群众竞技场。加快推进气象现代化建设，不断增强气象监测、预报、预警能力。加强国防动员、国防教育和国防后备力量建设，积极开展双拥工作，促进军民融合发展。加大公积金归集扩面力度。继续做好人防、消防、地震、统计、气象、档案、地方志、人口计生、应急管理、机关事务管理等工作，推动民族宗教、外事、侨务、对台、工会、老年人、残疾人、妇女儿童、青少年等事业发展。 
    坚持全覆盖、保基本、多层次、可持续的原则，加速完善就业服务体系，持续提升社会保障水平，着力筑牢民生之本，不断夯实和谐之基。鼓励以创业带动就业，加强政府公共就业服务能力，新增城镇就业6万人以上。持续开展社保扩面征缴专项行动，维护职工合法权益。适时调整城乡居民养老保险、被征地农民养老保险待遇水平。推进企业劳动保障诚信体系建设，构建和谐劳动关系。落实城市低保、大病救助、五保供养等政策。实行城乡居民临时救助，资助贫困大学生、困难家庭学龄前儿童入园，对城乡80岁、百岁以上老人给予补贴，实施一户多残家庭生活补助。推进精准扶贫，减少贫困人口2万人以上。 
    全面推进依法治市，深入开展多层次多领域依法治理，让办事依法、遇事找法、解决问题用法、化解矛盾靠法成为社会新风尚，最大限度增加社会和谐因素。深入开展公共法律服务体系建设，以法治思维、法治方式处理各类矛盾纠纷。研究制定示范社区建设标准，按照每个城市社区300平方米的规模，完成10个以上社区活动场所建设任务。完善突发公共事件应急处置机制，不断提高预防和处置各类突发公共事件能力。敬畏生命、敬畏责任、敬畏制度，坚持不懈抓好危化品、道路交通、消防、电梯等领域安全生产，强化食品药品安全监管，依法严厉打击各类刑事犯罪，坚决杜绝重大安全事故，坚决保障群众饮食用药安全，坚决维护社会大局和谐稳定。 
    三、勤勉尽责，新规则中锤炼新作风 
    各位代表，新常态、新机遇、新规则对政府工作和自身建设提出了新的更高要求，我们将以更加奋发有为的精神状态和勤勉务实的工作作风，持续提升创造力、战斗力、执行力、亲和力、抵抗力，着力建设为民、务实、清廉、高效政府。 
    （一）规范用权，依法行政。强化法治思维，践行法治理念，坚持法定职责必须为，法无授权不可为，坚决纠正不作为、乱作为，坚决惩处失职、渎职。坚持把公众参与、专家论证、风险评估、合法性审查、集体讨论决定确定为重大行政决策法定程序，建立重大决策终身责任追究制度和责任倒查机制。深化行政执法体制改革，推进综合执法，严格实行行政执法人员持证上岗和资格管理制度。坚持以公开为常态、不公开为例外原则，梳理公布政府权力清单和责任清单，及时发布财政预决算、公共资源配置、重大建设项目批准和实施等领域信息。自觉接受市人大法律监督、市政协民主监督和社会公众舆论监督，定期向市人大及其常委会报告工作，向市政协通报情况，重大决策、重要政策、重点工作在论证中，及时认真地听取各民主党派、工商联、无党派人士和人民团体的意见。高效办理人大代表议案、建议和政协委员提案，支持、配合人大代表、政协委员对政府重点、难点工作开展调研、视察和专题询问、协商。 
    （二）勇于创新，科学理政。始终把新常态理念贯穿于经济社会发展各个环节，更加重视科学性研判、前瞻性考虑、可行性分析、系统性运作，善于向市场要资源、要活力、要手段。以“功成不必在我”的胸襟和定力，一件件理出打基础、利长远的发展目标，一项项破解制约重大项目推进的根本性问题。坚持把改革创新作为政府工作的永恒主题和不竭动力，以改革攻坚破除体制机制旧羁绊，以创新驱动构筑科学发展新根基，在严峻挑战中抓住机遇，在激烈竞争中争取主动。深入推进学习型政府建设，注重学习政策、研究政策、用好政策，把握经济运行新形势，适应宏观调控新要求。突出“四化同步”、“五位一体”理念与各县区实践紧密结合，优化目标考核评价体系，彰显区域功能特征，明确差异分工任务，增强科学性、针对性和实效性。 
    （三）转变职能，亲民施政。按照“集中、便民、提效”的要求，以更大气力把简政放权、放管结合改革推向深入，切实把该放的放到位，把该给的给到位，把该管的管到位，保障市场公平有序竞争，以政府自身革命带动重要领域改革。积极稳妥推进事业单位分类改革，扩大政府购买公共服务范围。把群众满意度、幸福感作为政府工作成效的标度尺，着力研究群众关心的事，做好群众盼望的事，整改群众不满的事，多办群众受益的事。深入实施服务企业、服务百姓“百千万”行动，让企业经营安心，让客商投资放心，让百姓生活舒心。探索建立跨区域社区综合性服务中心，将相关职能部门面向基层和群众的公共服务事项委托社区承担，解决好联系服务群众“最后一公里”、“最后一步路”问题。做优市长热线服务中心、政风行风热线等平台，畅通诉求渠道，问计民生、汇聚民意、纾解民困。 
    （四）求真务实，高效勤政。坚定奋力崛起的信心，鼓足赶超跨越的干劲，主动发出“好声音”，积极释放“正能量”。大力弘扬钉钉子精神，强化“我负责、我来办、马上办”理念，对每一个重大项目、每一项重点工作，都细化到“干什么、怎么干、谁来干、什么时间干成、干不成了怎么办”，养成人人讲效率、事事快节奏、件件求实效作风习惯。加强对重大决策、重要工作、重点项目的督查和调度，构建便捷高效的工作督查机制，工作部署到哪里，督查就跟踪到哪里，工作不完成，督查不停止。继续大力精文简会，创新工作部署方式，腾出更多时间和精力下基层、解难题、促发展。强化干部问责、问绩、问效措施，坚决克服等待观望和畏难情绪，坚决纠正懒政、怠政和为官不为现象，坚决摒弃袖手旁观和操手议论，让想干事的有机会，能干事的有舞台，干成事的更有为。 
    （五）筑牢防线，廉洁从政。坚决贯彻中央“八项规定”、国务院“约法三章”和省、市作风建设各项规定，在遵纪守法中做好工作，在严格律己中担当责任，在规矩约束下干事创业，守住底线、不闯红线。巩固扩大群众路线教育实践活动成果，严防“四风”问题反弹、回潮，推动作风建设常态化、长效化。严格落实党风廉政建设责任制，健全惩治和预防腐败体系，坚持用制度管权管事管人，努力形成不敢腐的惩戒机制、不能腐的防范机制、不易腐的保障机制。强化效能监察和审计结果运用，确保政令畅通，推动各项工作提速增效。牢固树立过紧日子思想，严控“三公”经费支出，推进公务用车制度改革，大力削减行政成本，把有限的资源和财力更多用在发展经济和改善民生上。 
    各位代表，赶超跨越正当其时，拼搏实干定能圆梦。让我们在中共蚌埠市委的坚强领导下，紧紧依靠全市人民，坚定信心，攻坚克难，扎实苦干，圆满完成“十二五”目标任务，为全面建成小康社会、重返全省第一方阵再努力、再冲刺、再奋斗！
